# Median Lingual Foramen, a new midmandibular cephalometric landmark

**DOI:** 10.1111/ocr.12372

**Published:** 2020-03-10

**Authors:** David Vandekerckhove, Dionne Deibel, Shankeeth Vinayahalingam, Gilles Claeys, Tae‐Geon Kwon, Stefaan Bergé, Tong Xi

**Affiliations:** ^1^ Department of Oral and Maxillofacial Surgery Radboud University Nijmegen Medical Centre Nijmegen The Netherlands; ^2^ Department of Oral and Maxillofacial Surgery School of Dentistry Kyungpook National University Daegu Korea

**Keywords:** asymmetric, cephalometric landmark, mandible, orthognathic surgery

## Abstract

**Purpose:**

In asymmetrical mandibles, it is often challenging to identify the mandibular midline. The median lingual foramen (MLF) is located at the midline of the anterior mandible. The purpose of this study is to evaluate the reproducibility of identifying the MLF compared to conventional landmarks on cone beam computed tomography's (CBCT's) to mark the mandibular midline.

**Material and Methods:**

Ten symmetrical class II, 10 symmetrical class III, ten asymmetrical class II and 10 asymmetrical class III patients were included. On CBCTs, the cephalometric landmarks menton, pogonion, genial tubercle and MLF were identified twice by two observers.

**Results:**

A high intra‐ and interobserver reproducibility was found for all landmarks, the highest being the MLF. The gain in accuracy is 0.998 mm, 0.824 mm and 0.361 mm compared to pogonion, genial tubercle and menton, respectively (*P*‐value <.05).

**Conclusion:**

MLF is a reliable and reproducible landmark to indicate the midline of the mandible, particularly in Class II asymmetric mandibles.

## 1 INTRODUCTION

Surgical correction of mandibular asymmetry is challenging in the field of orthognathic surgery. The aberrant shape as well as the malposition of the mandible results in multiplanar facial asymmetries.^1^

The emergence of three‐dimensional (3D) cone beam computed tomography (CBCT) has enabled orthognathic surgeons to diagnose maxillofacial deformities and to allow a more accurate pre‐operative planning.^1^, ^2^, ^3^, ^4^, ^5^, ^6^ Whilst planning an operation, cephalometric landmarks are used to determine the morphology of the facial skeleton. The traditional landmarks to indicate the midline of the mandible include pogonion, mention and genial tubercle. The reliability and reproducibility of conventional cephalometric landmarks were validated on patients without a clinically significant asymmetry of the mandible.^3^, ^7^, ^8^, ^9^, ^10^ In asymmetrical cases, the landmarks are more difficult to identify.

Mirroring is frequently used to superimpose one hemimandible upon the other in order to analyse the severity of mandibular asymmetries and calculate the difference in volume. It provides accurate guidance throughout the surgery, minimalizing the residual asymmetry.^11^, ^12^ The most challenging issue in mirroring is the selection of the mirroring plane, or the midline of the mandible.^4^, ^13^

Whilst analysing CBCT data in our daily practice, a distinct anatomical structure was noticed in the mandibular midline of the symphysis region, which seemed to be universally present. Upon reviewing literature, this anatomical landmark was named the median (or midline) lingual canal (MLC), which had previously been describes.^14^, ^15^, ^16^, ^17^

Inferior to the apices of the lower central incisors and in some cases just superior to the genial tubercles, a neurovascular bundle perforates the lingual cortex of the mandible, called the medial lingual foramen (MLF). Even though the vertical location of the MLF varies, it is always located at the midline of the mandible on the transverse plane. Therefore, the MLF has the potential to be used as a landmark for patients with asymmetrical mandibles.^18^

The aim of this study is to evaluate the reproducible and reliability of MLF as a cephalometric landmark compared to traditional landmarks.

## 2 MATERIALS AND METHODS

### 2.1 Subjects

CBCT images of patients who visited the Department of Oral and Maxillofacial Surgery at the Radboud University Nijmegen Medical Centre, the Netherlands or the Department of Oral and Maxillofacial Surgery at the affiliated hospital of Kyungpook National University in Daegu, South Korea, and who consented to CBCT imaging as part of the diagnostic evaluation, were eligible for this study. We stratified the patient intake. Forty patients were selected for this study, 10 patients with symmetrical class II, 10 patients with asymmetric class II, 10 patients with symmetrical class III and 10 patients with asymmetric class III skeletal relationship. The inclusion of asymmetric patients was defined by the presence of transverse midline deviation of more than 3mm measured at the menton. The enrolment of retrognathic patients was characterized by the ANB >4° whilst the selection of prognathic patients was based on ANB <0°. Patients with class I skeletal jaw relationship, syndromic patients and patients with facial trauma in their medical history were excluded. All data were anonymized and de‐identified prior to analysis. Informed consent was waived by the Institutional Review Board due to the retrospective nature of the study. The estimated sample size of N = 10 is in line with other comparable landmark studies.^8^

### 2.2 Imaging methods and set‐up of reference frame

Of all patients, a cone beam CT (CBCT) of the entire mandible was available, acquired using standardized CBCT scanning protocols, FOV 23 cm diameter/17 cm height, scan time 17,8 seconds, voxel size 0.4 mm, at 120 kVp, 37.1 mAs at the Radboud University Medical Center and using FOV 19 cm diameter/19 cm height, scan time 9.6 seconds, voxel size 0.4 mm, at 120 kVp, 15 mAs (CB MercuRay CBCT scanner; Hitachi Medical Systems) at the affiliated hospital of Kyungpook National University in Daegu. Scans of low quality due to scattering or movement artefacts were excluded. After visual selection of 40 patients by a first observer, a second observer confirmed whether or not all subjects met the inclusion criteria.

The DICOM files (Digital Imaging and Communications in Medicine) were imported in Maxilim^®^ software (Medicim NV), and a 3D hard‐tissue surface model was rendered.

First a reference frame was set‐up using the landmarks sella and nasion to reconstruct the horizontal, median and vertical reference planes.^10^ Also, three axes were set‐up, having the X‐axis directed from left to right, the Y‐axis from front to back and the Z‐axis from cranial to caudal.

### 2.3 Landmark identification

The traditional cephalometric landmarks pogonion, menton and genial tubercle were identified by the observer. The definition of the landmarks and cephalometric planes used are shown in Table 1.

**Table 1:** Definition of cephalometric landmarks and planes used in this study

Landmarks	Abbreviation	Definition
Nasion	N	The midpoint of the frontonasal suture
Sella	S	The centre of the hypophyseal fossa (sella turcica)
Horizontal (*x*‐*y*) 3‐D Cephalometric Reference Plane	HP	A plane 6 degrees below the Anterior Cranial Base (S‐N) plane, through Sella and along the horizontal direction of the natural head position
Median (*z*‐*y*) 3‐D Cephalometric Reference Plane	MP	A plane through Sella and Nasion and perpendicular to the Horizontal 3‐D Cephalometric Reference Plane
Vertical (*x*‐*z*) 3‐D Cephalometric Reference Plane	VP	A plane through Nasion and perpendicular to the Horizontal and Median 3‐D Cephalometric Reference Plane
Pogonion	Pog	The most projecting median point on the anterior surface of the chin
Menton	Me	The most inferior part in the middle of the bony chin.
Genial Tubercle	GT	The middle of the eminence of bone found on the lingual side of the mandible
Median Lingual Foramen	MLF	The junction between the lingual cortical bone of the anterior mandible and the cranial bone surrounding the radiolucent canal perforating the lingual cortex

The new landmark MLF was subsequently identified according to a well‐defined three‐step procedure.

**Figure 1:**
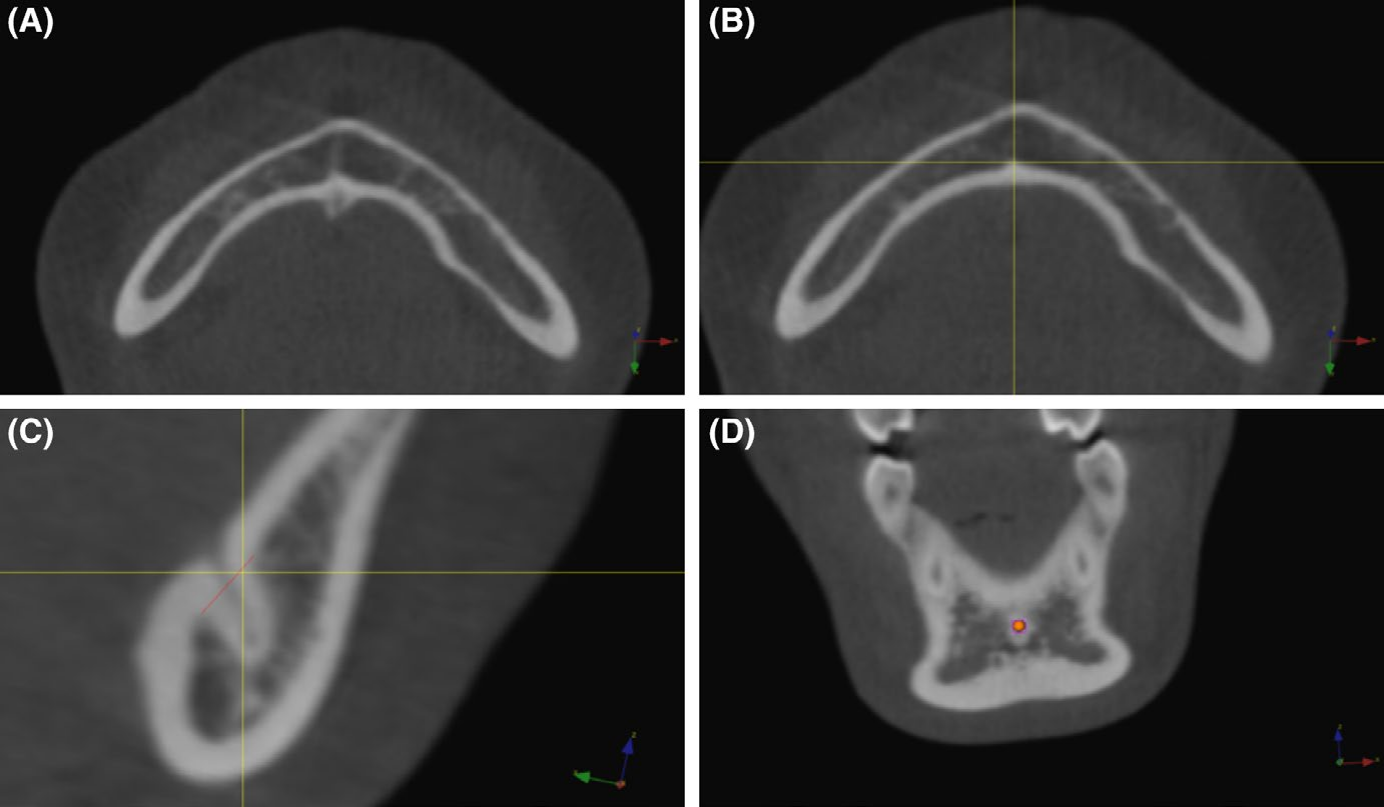
A, (upper left): This illustration displayed a radiolucent canal perforating the lingual side of the cortex in the midsagittal region of the mandible. Note the cortical outline which is situated towards the cancellous bone that surrounds the canal. B, (upper right): Median Lingual Foramen (MLF) is first plotted on the most cranial axial slice that showed an irregular form of the lingual cortex. C, (lower left): The sagittal slice that dissected the previously plotted landmark is selected. MLF is replotted at the junction of the lingual cortical bone of the mandible and the most cranial bone surrounding the radiolucent canal. The red line is constructed to aid the identification of the MLF. Whenever there is more than one canal present, the superior canal will be used (MLFsu) as opposed to the inferior canal (MLFinf). D, (lower right): As a final step, the position of MLF is checked on the coronal slice

MLF is visualized by scrolling through the axial slices in a cranio‐caudal direction. In the region below the apices of the inferior incisors, a small radiolucent canal is present perforating the lingual cortex (Figure 1A). When two or more canals were present, the more superior canal (MLFsu) was used. The MLF landmark was plotted on the most cranial slice, on which the lingual cortex showed an irregular form (Figure 1B).

The sagittal slice that dissected the plotted MLF was selected to maintain its position in the medio‐lateral direction, that is, the x‐axis. The MLF landmark is replotted at the intersection between the lingual cortical bone of the mandible and the most cranial bone surrounding the radiolucent canal. In this way, the position of the MLF on the y‐ and z‐axis can be determined. To aid the identification of MLF in this final step, a line can be drawn connecting the lingual cortical bone cranially and caudally to the radiolucent canal (Figure 1C).

Finally, the MLF landmark is checked on the corresponding coronal slice (Figure 1D). In case the correct position was questioned, the three‐step procedure was repeated.

All four landmarks (pogonion, menton, genial tubercle and MLF) were identified twice on each mandible by the first observer with a time interval of a minimum of three weeks to prevent memory bias. A second observer identified the four landmarks on all CBCT scans once.

After completing the landmark identification, cephalometric measurements comprising the distances from each point to the horizontal, vertical and median planes were computed and exported to Microsoft Office Excel 2007^®^ (Microsoft Corporation) for further analysis.

### 2.4 Statistical analysis

The statistical data analysis was carried out with the SPSS software program, version 22 for windows (SPSS Inc) by a professional statistician. The discrepancy between the observers in plotting landmarks was calculated using the 3D Euclidean distance, which represents the distance between two points in space (three dimensions). The Euclidean distance between two landmarks for example A1 (Xa1, Ya1, Za1) and A2 (Xa2, Ya2, Za2) was calculated with the formula $\sqrt{(\left(\text{Xa}1-{\text{Xa2)}}^{2}+{\left[{\left(\text{Ya}1-\text{Ya}2\right)}^{2}+\left(\text{Za}1-\text{Za}2\right)\right]}^{2}\right)}$. The comparison of Euclidean distance or individual coordinates within or between observers, was done with paired t‐tests. The duplicate measurement error was calculated as the standard deviation of the differences divided by $\sqrt{2}$. The reliability coefficient was calculated using the Pearson correlation coefficient. The comparison of interobserver differences found with various landmarks, was done using *t*‐test.

## 3 RESULTS

An intra‐ and interobserver reliability of 0.978 or more was calculated for all cephalometric landmarks, indicating a steady and predictable way of plotting by the observers.

**Figure 2:**
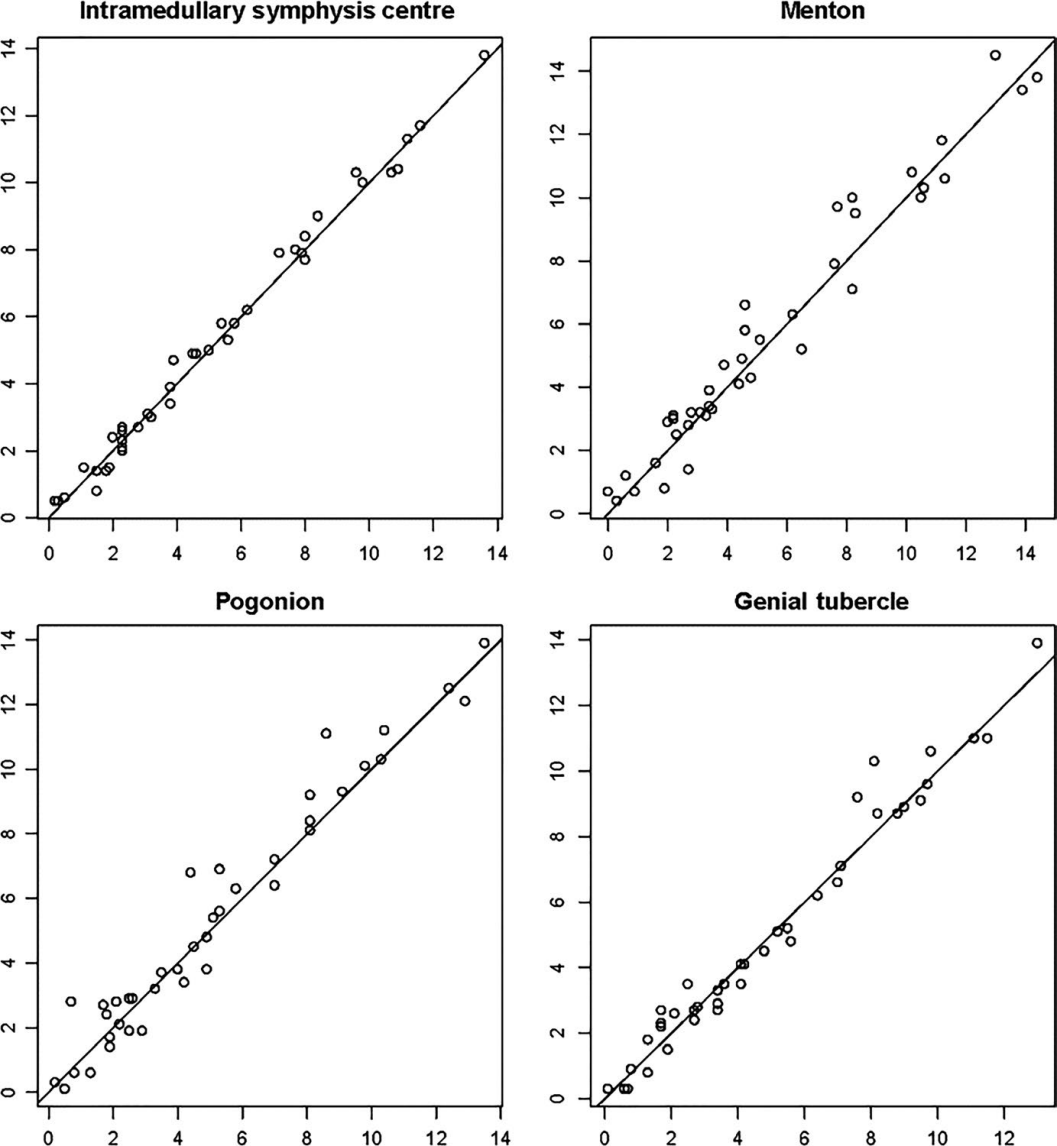
Scatterplots showing the interobserver agreement in the latero‐lateral dimension (*X*‐axis) for Median Lingual Foramen, Menton, Pogonion and Genial Tubercle. The closer the dots situated to the diagonal, the higher the agreement

A scatterplot for the interobserver agreement of the landmarks MLF, menton, pogonion and genial tubercle on the x‐axis (transverse plane) was constructed (Figure 2). This scatterplot illustrated a good interobserver agreement of all landmarks, MLF in particular.

The interobserver discrepancy or Euclidean distance was calculated for all traditional landmarks (Me: 1.152 mm, Pog: 1.789 mm, GT: 1.615 mm) and MLF (0.790 mm). The Euclidean distance calculated for MLF was subtracted from the Euclidean distances calculated for the traditional landmark, exhibiting the increase in accuracy of MLF over traditional landmarks. A positive result indicated a smaller interobserver discrepancy for MLF and hence a better performance on MLF. A negative result indicated a greater interobserver discrepancy for MLF and hence a better performance on traditional landmarks. The results showed a gain in accuracy of MLF compared to the traditional landmarks: 0.361 mm compared to menton (*P* = .010), 0.988 mm compared to pogonion (*P* = .004) and 0.824 mm compared to genial tubercle (*P* = .013).

Subsequently, an identical analysis was performed to evaluate the interobserver discrepancy in the latero‐lateral (*X*‐axis), antero‐posterior (*Y*‐axis) and cranio‐caudal (*Z*‐axis) directions. The interobserver discrepancy was smaller for MLF compared to all traditional landmarks except for Menton in the antero‐posterior direction.

To investigate the influence of asymmetry and jaw relationship (class II/III) on the interobserver discrepancy, a similar analysis was performed on the skeletal subgroups. The results showed a significant statistical increase in accuracy of MLF in all class II asymmetrical mandibles (Me: 0.803 mm, Pog: 1.579 mm, GT: 1.314 mm), and a gain in class III asymmetrical mandibles as well (Me: 0.397 mm (*P* = .08), Pog: 0.775 mm (*P* = .17), GT: 1.314 mm (*P* < .05).

The interobserver discrepancy was smaller for MLF compared to all landmarks for all skeletal relationships, except for Menton in class III symmetrical patients (0.081 mm (*P* = .716). This superior performance of MLF was more pronounced in asymmetric and class II patients as the differences (the Euclidean distance of traditional landmarks minus Euclidean distance MLF) were greater and the p‐values smaller.

## 4 DISCUSSION

This study has demonstrated a protocolized method to identify the anatomical landmark MLF on CBCTs and has shown that MLF is a reproducible landmark to indicate the transverse midline of the mandible. To accomplish high intra‐ and interobserver reliability, a clear description of the steps to identify the MLF was developed. The identification of MLF is straightforward, unambiguous and efficient.

A possible challenge of identifying the MLF may be the quality of the CBCT scan, as illustrated by the difference in the occurrence in cadaver and patient studies and studies where CBCT was used.^17^ In one patient, the MLC was visible on the axial slice, but not on the sagittal slice, due to small diameter of the foramen. In another patient, multiple exostoses on the lingual side of the mandible, in combination with dense cancellous bone, caused difficulties in identifying the location of the neurovascular bundle. Despite these inconveniences, we were able to plot MLF in every patient by using two different CBCT scanners and scanning protocols.

The interobserver discrepancy was smallest for MLF compared to the other traditional landmarks. This increase in accuracy is believed to be explained by the use of multiplanar CBCT slices instead of using a 3D rendered surface model.^9^ In addition, MLF is based on a small and distinct anatomical structure instead of a broader surface, its identification is less influenced by the view angle and the magnitude of mandibular asymmetry.

A greater increase in accuracy in the identification of MLF was found among patients with mandibular asymmetry. As the location of a midmandibular landmark in the x‐axis is the most clinically relevant among patients with mandibular asymmetry, the use of MLF would ease the pre‐operative planning and post‐operative evaluation of orthognathic surgery, as it is able to indicate the true anatomical centre of an (asymmetric) mandible.^1^, ^13^, ^18^, ^19^, ^20^

Further studies might be required in order to assess the correlation between MLF and different jaw deformities. In addition, the MLF might be helpful in the classification of different mandibular asymmetries.

## 5 CONCLUSION

The present study demonstrates that the new anatomical cephalometric landmark MLF can be identified in a more accurate, easier and reproducible way compared to conventional midline cephalometric landmarks.

## CONFLICT OF INTEREST

The authors have no conflict of interest to declare.
